# Durable Long-Term Bacterial Engraftment following Encapsulated Fecal Microbiota Transplantation To Treat Clostridium difficile Infection

**DOI:** 10.1128/mBio.01586-19

**Published:** 2019-07-23

**Authors:** Christopher Staley, Thomas Kaiser, Byron P. Vaughn, Carolyn Graiziger, Matthew J. Hamilton, Amanda J. Kabage, Alexander Khoruts, Michael J. Sadowsky

**Affiliations:** a Division of Basic & Translational Research, Department of Surgery, University of Minnesota, Minneapolis, Minnesota, USA; b BioTechnology Institute, University of Minnesota, Saint Paul, Minnesota, USA; c Division of Gastroenterology, Department of Medicine, University of Minnesota, Minneapolis, Minnesota, USA; d Department of Soil, Water, and Climate, University of Minnesota, Saint Paul, Minnesota, USA; e Department of Plant and Microbial Biology, University of Minnesota, Saint Paul, Minnesota, USA; Yale School of Public Health; Pacific Northwest National Laboratory; University of Maryland, School of Medicine

**Keywords:** *Bacteroides*, capsule FMT, donor, engraftment, fecal transplant, stable

## Abstract

Fecal microbiota transplantation (FMT) has become a common rescue therapy for recurrent Clostridium difficile infection, and encapsulated delivery (cFMT) of healthy donor microbiota shows similar clinical efficacy as more traditional routes of administration. In this study, we characterized long-term patterns of bacterial engraftment in a cohort of 18 patients, who received capsules from one of three donors, up to 409 days post-FMT. Bacterial communities were characterized using Illumina sequencing of the V5-V6 hypervariable regions of the 16S rRNA gene, and engraftment was determined by using the Bayesian algorithm SourceTracker. All patients recovered clinically and were free of C. difficile infection following cFMT. The majority of patients (61%) showed high levels of engraftment after the first week following FMT, which were sustained throughout the year. A small subset, 22%, experienced a decline in donor engraftment after approximately 1 month, and a few patients (17%), two of whom were taking metformin, showed delayed and low levels of donor engraftment. Members of the genera *Bacteroides*, *Parabacteroides*, and *Faecalibacterium* were significantly and positively correlated with donor similarity (ρ = 0.237 to 0.373, *P* ≤ 0.017). Furthermore, throughout the year, patient fecal communities showed significant separation based on the donor fecal microbiota that they received (*P* < 0.001). Results of this study, which characterize long-term engraftment following cFMT, suggest that numerical donor similarity is not strictly related to clinical outcome and identify a persistent donor-specific effect on patient fecal microbial communities. Furthermore, results suggest that members of the *Bacteroidetes* may be important targets to improve engraftment via cFMT.

## Abstract (precis)

Recurrent Clostridium difficile infection (rCDI) is the most common cause of hospital- and community-acquired diarrheal infection associated with antibiotic use. Fecal microbiota transplantation (FMT), a treatment that involves administration of fecal bacteria from a healthy donor to a recipient patient, is a highly effective rescue therapy for rCDI that is increasingly being incorporated into standard clinical practice. Encapsulated, freeze-dried preparations of fecal microbiota, administered orally, offer the simplest and most convenient route of FMT delivery for patients (cFMT). In this study, we evaluated the extent of bacterial engraftment following cFMT and the duration of donor bacterial persistence. All patients studied recovered clinically but showed differing patterns in long-term microbial community similarity to the donor that were associated with members of the bacterial group *Bacteroidetes*, previously shown to be prominent contributors to rCDI resistance. Results highlight long-lasting, donor-specific effects on recipient patient microbiota and reveal potential bacterial targets to improve cFMT engraftment.

## INTRODUCTION

Suppression of the intestinal microbiota, predominantly through the use of antibiotics, results in decreased colonization resistance and can lead to infection by Clostridium difficile (1). The incidence, morbidity, and mortality associated with C. difficile infections have risen over the past 2 decades, creating a major burden on the health care system (2, 3). Recurrent C. difficile infections (rCDIs), in particular, present a major clinical problem and continue to increase in incidence (4). Fecal microbiota transplantation (FMT), an approach that aims to restore normal intestinal microbial community structure following antibiotic-induced damage, has become standard rescue therapy to treat rCDI (5). The treatment shows a high rate of clinical success, approaching 90% (6, 7), and can be performed using microbiota from stool of prescreened, healthy donors (8, 9). Furthermore, the fecal microbiota preparations may be encapsulated for easier administration (10, 11). Capsule delivery (cFMT) has shown similar clinical results as colonoscopic administration in a randomized clinical trial (12). However, while colonoscopic delivery results in a restoration of a healthy microbiota composition within several days (13), delivery of microbiota by capsule results in punctuated changes in the composition of the microbiota, taking several weeks to taxonomically resemble a donor-like, intestinal microbial community despite resolution of clinical symptoms (14).

There remains considerable uncertainty with regard to the stability of post-FMT microbiota. In a four-patient study with dense sampling, we observed some divergence in taxonomic composition of the initial, post-engraftment microbiota away from the donor sample over up to a 5-month period following colonoscopic FMT (13). However, these shifts were similar to the normal temporal variation observed in healthy donor samples. Similarly, a single-patient report of a recovered rCDI patient showed compositional fluctuations through 7 months following FMT (15), with further normalization to resemble order-level donor microbiota composition after 4.5 years (16). A similar study of 14 rCDI patients noted that the patient microbiota composition following FMT was more highly correlated with those of donors (*x̄* = 95.3%) than would be expected given normal, interpersonal variability (*x̄* = 77.4%), and engraftment persisted through a 1-year follow-up (17). Robust characterization of individualized donor bacterial engraftment patterns, beyond observational remarks or correlative analysis, however, has not been evaluated in long-term studies. Furthermore, key taxa that promote bacterial engraftment and resolution of clinical symptoms have not been conclusively described.

We previously reported that cFMT causes incremental shifts in the intestinal microbiota composition, with stepwise progression toward a community that taxonomically resembles that of the donor (14). We also observed that bacterial communities following cFMT showed greater similarity to microbiota from a pool of healthy donors, rather than to the microbiota of their specific donor (18), suggesting that cFMT may result in healthy bacterial community reorganization, independently of specific donor similarity. We focused these initial studies on the early post-treatment events because the clinical efficacy of FMT in breaking the cycle of CDI recurrence is typically assessed at ∼2 months following treatment. However, a substantial fraction of rCDI patients treated with FMT remain vulnerable to CDI relapse with new antibiotic provocations (19, 20), an observation that raises concerns about the stability and resilience of the post-FMT microbiota. While we observed that the donor bacterial engraftment was stable through a 2-month follow-up time point, the extent of sustained, longer-term engraftment has thus far not been evaluated. Thus, the goals of this study were to assess long-term (over 1 year) patterns of engraftment in a subset of patients who responded to cFMT without recurrence of C. difficile infection or new antibiotic exposure. We further aimed to clarify the relationship between the empirical donor microbiota and the relative return of the patient microbiota to a “healthy” assemblage reflected by multiple individual donors. We hypothesized that, similarly to FMT administered colonoscopically, patients would show minor divergence from the donor but otherwise sustain high levels of engraftment. Results of this study reveal various patterns in long-term bacterial engraftment following cFMT, independent of clinical recovery. We further highlight the potential of species within the genus *Bacteroides* to serve as a keystone species in maintaining the donor bacterial signature.

## RESULTS

### Patient cohort.

The microbiome was characterized over a period of at least 1 year from stool samples of 20 patients (Table 1). Patients received encapsulated, lyophilized donor fecal microbiota delivered orally. Of these, nine received capsules prepared from donor 6, 10 received capsules from donor 41, and one received capsules from donor 44. Two patients, one who received capsules prepared from donor 6 and the other from donor 41, experienced a recurrence of infection following the initial cFMT and were excluded from downstream analyses. Samples were grouped by categorical time points including samples collected prior to cFMT (pre-FMT) and at the following time periods after cFMT: 2 to 6 days, 7 to 21 days, 28 to 45 days, 86 to 134 days, and 346 to 409 days. This is similar to our previous reports (14, 18) and corresponds to early incremental shifts in the microbiota.

**TABLE 1:** Demographics and clinical characteristics of the patients included in the stool analysis study

Demographic or clinical characteristic	Value
Age, yr (mean ± SD)	54 ± 13
Sex (% female)	72
History of hospitalization for severe or fulminant *C. difficile* infection (%)	33
Median no. of mo between the initial *C. difficile* infection and cFMT treatment (range)	9.5 (4–70)
Use of proton pump inhibitors (%)	11
Use of statin medications (%)	44
Use of metformin (%)	11
Body mass index, kg m^−2^ (mean ± SD)	26.5 ± 8.4

### Microbial community diversity and composition.

**FIG 1:**
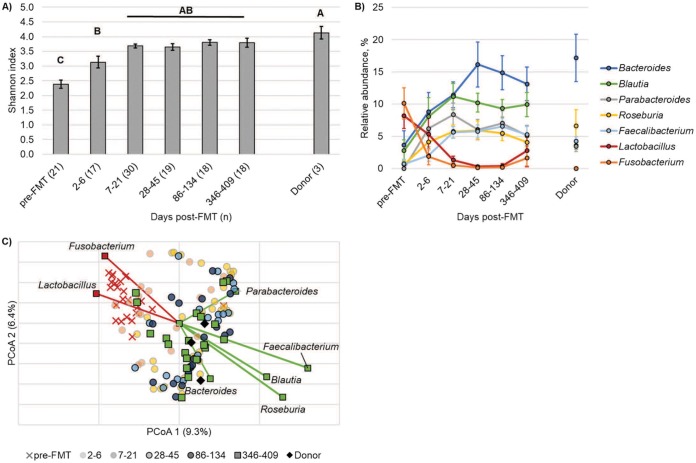
Microbial community diversity and composition in patient and donor samples. (A) Shannon indices in samples grouped by time point. Error bars reflect standard error, letters denote statistical differences (Tukey’s *post hoc P* < 0.05), and bars that share the same letter did not differ significantly. (B) Distribution of abundant genera in samples grouped by time point (mean ± standard error). (C) Principal-coordinate analysis of Bray-Curtis distances among all patient and donor samples (*r*^2^ = 0.40). Samples are grouped by days post-FMT, pre-FMT, or donor. Abundant genera that were significantly correlated with axis positions are shown.

Incremental increases in diversity and bacterial community composition were observed in patient samples following cFMT, with a return to a donor-like assemblage after the first week. A mean Good’s coverage estimate of 99.0% ± 0.6% was achieved among all samples. Alpha diversity, characterized by using the Shannon index, was significantly lower in pre-FMT samples and increased significantly at 2 to 6 days post-FMT (*post hoc P* = 0.001; Fig. 1A). By the first week following cFMT, alpha diversity recovered to a similar level as that observed in donor samples and was maintained throughout the year. Similarly to alpha diversity, bacterial community composition differed significantly in the pre-FMT samples (analysis of similarity [ANOSIM] *R* = 0.18 to 0.85, *P* < 0.001), changed significantly at 2 to 6 days post-FMT (*R* = 0.18, *P* < 0.001), and was not statistically different from donor samples after the first week (*R* ≤ 0.22, *P* ≥ 0.035 at Bonferroni corrected α = 0.002; Fig. 1B). Changes in microbial (bacterial) community composition were predominantly characterized by decreases in members of the genera *Fusobacterium* and *Lactobacillus* and increases in relative abundances of *Bacteroides*, *Blautia*, *Parabacteroides*, *Roseburia*, and *Faecalibacterium* (Fig. 1C).

### Duration of microbial engraftment.

**FIG 2:**
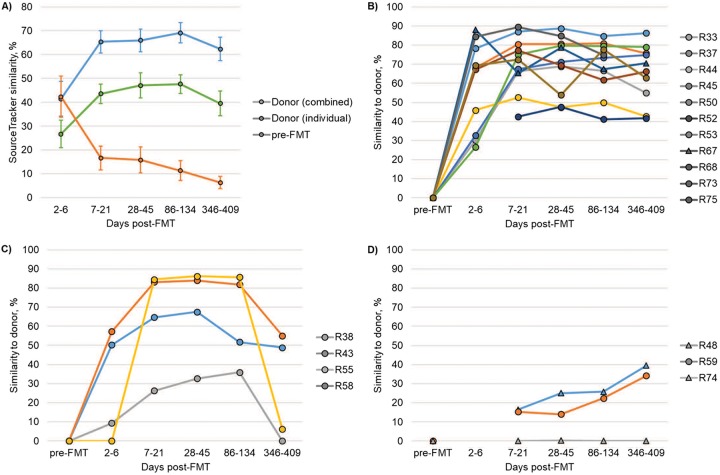
Evaluation of donor engraftment in patient samples. (A) Mean similarity to all three donor lots (combined donor), the donor lot received (individual donor), or the patient’s pre-FMT sample, as determined by SourceTracker. (B) Patients showing sustained engraftment through the year-long follow-up. (C) Patients in whom a decline in donor similarity (approximately 20% or greater from maximum) was observed. (D) Patients in whom slow and/or poor engraftment was observed. For panels B to D, all data points were normalized to donor similarity in the patient’s pre-FMT sample and only the donor lot received was used as a source. Missing time points indicate that a sample was not collected during that period. Patients who were taking metformin are shown by triangles. Error bars reflect standard error.

SourceTracker software, which uses a Bayesian algorithm to determine the percentage of the community in user-defined sink (patient) samples that is attributable to source (donor) communities, was used to measure bacterial engraftment. The percentage of donor engraftment, from individual donor lots, increased significantly from days 2 to 6 to days 28 to 45 post-FMT (*post hoc P* = 0.026; Fig. 2A) but did not change significantly following the first weeks after FMT (*P* ≥ 0.739). However, significantly greater similarity was observed when all three donors were assigned as a single potential source (“combined donor”) than when only the empirically administered donor (“individual donor”) sample was used (*P* < 0.0001).

Taxa primarily contributing to engraftment that persisted throughout the year were predominantly classified within the genera *Bacteroides* (12.27% ± 0.03% of sequence reads), *Blautia* (5.52% ± 0.01%), *Parabacteroides* (4.29% ± 0.01%), *Roseburia* (3.30% ± 0.01), and *Faecalibacterium* (2.95% ± 0.01%), corresponding to changes observed in community composition over time (Fig. 1B). Taxa within less-abundant genera (23.32% ± 0.03%), however, also contributed considerably to donor engraftment predictions using SourceTracker.

The extent and duration of engraftment varied by individual patient (Fig. 2B to D), with three general patterns observed. The majority of patients (61.1%, 11 patients) showed an increase in donor similarity after the first week post-FMT, and the extent of donor similarity remained relatively stable throughout the year (Fig. 2B). A smaller group of patients (22.2%, 4 patients) showed an increase in donor similarity through the first month, followed by a sharp decline (≥20% decline from maximum similarity) at the end of the year (Fig. 2C). A minority of patients (16.7%, 3 patients) experienced low levels of engraftment (<50% donor similarity) that occurred slowly, reaching a maximum similarity in 1-year samples (Fig. 2D). Interestingly, two of three patients taking metformin fell into this final group, with one (R74) showing no substantial increase in donor similarity following cFMT. No patients experienced a spontaneous recurrence of rCDI or gastrointestinal symptoms (e.g., diarrhea), regardless of engraftment pattern.

**FIG 3:**
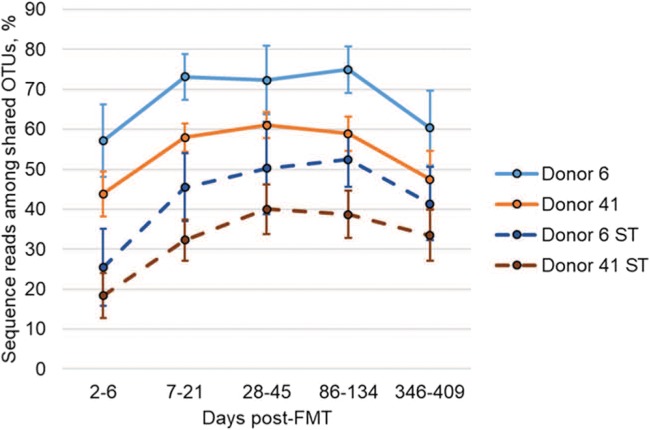
Similarity to donor samples using shared OTUs. Solid lines reflect the relative abundances of empirically shared OTUs. Dashed lines reflect SourceTracker (ST) predictions of donor similarity. Error bars reflect SEM.

In order to validate SourceTracker results, donor similarity was also determined using the abundances of operational taxonomic units (OTUs) that were empirically shared between donor 6 or 41 and patient samples (Fig. 3). While the percentage of sequences belonging to shared OTUs, among all patient samples receiving capsules from either donor, was significantly greater than donor engraftment as determined by SourceTracker (*P* < 0.0001), results from both methods of analysis were highly correlated (ρ = 0.837, *P* < 0.0001). A similar percentage of shared OTUs was observed among patients who received capsules from either donor 6 or donor 41, although communities in patient samples who received capsules from donor 6 showed a greater percentage of shared OTUs at 7 to 21 and 86 to 134 days post-FMT (*P* = 0.018 and 0.028, respectively).

### Maintenance of donor taxonomic signatures.

**FIG 4:**
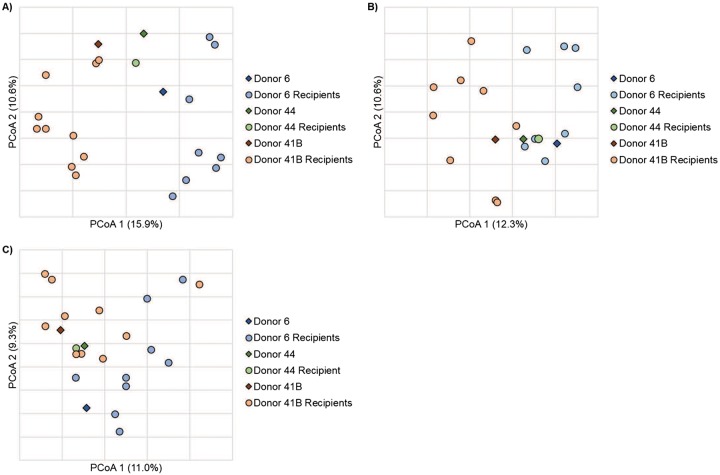
Principal-coordinate analysis of Bray-Curtis distances among patient samples collected: 28 to 45 days (*r*^2^ = 0.38) (A), 86 to 134 (*r*^2^ = 0.21) (B), and 346 to 409 days (*r*^2^ = 0.35) (C) post-FMT. Samples are grouped by the donor lot received.

The donor production lot (from a single stool) used for capsule preparation did not significantly influence the percentage of engraftment at the year-end time point (analysis of variance [ANOVA], *P* = 0.599). However, microbial communities from patient samples collected at least 1 month post-FMT showed similar composition as that of the donor lot that the patients received and were significantly different from patients receiving lots from other donors (ANOSIM *R* = 0.38, *P < *0.001; Fig. 4). Among individual patients, a greater alpha diversity in pre-FMT samples tended to negatively impact donor engraftment through the year-end time point, although this association was not significant (Spearman’s ρ = −0.266, *P* = 0.337).

**FIG 5:**
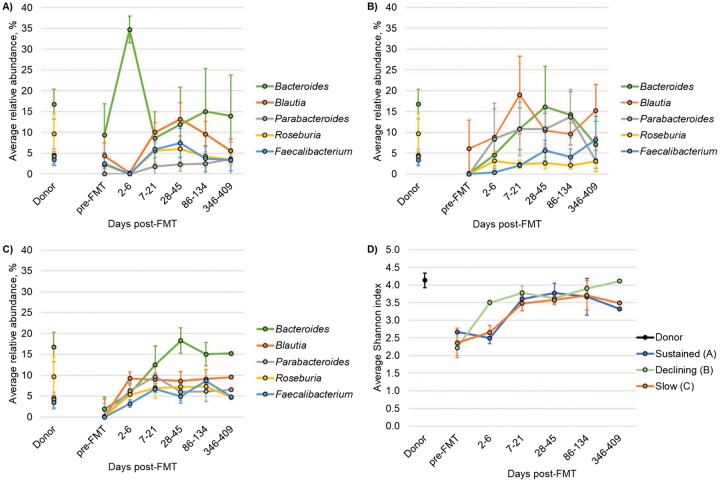
Bacterial community composition and diversity grouped by engraftment patterns. (A to C) Mean relative abundances of the five predominant genera are shown for samples from patients with sustained engraftment (A), declining engraftment (B), or slow/poor engraftment (C). (D) Mean Shannon indices for each engraftment pattern. Error bars reflect SEM.

Taxonomic changes were evaluated based on the three engraftment patterns observed. Patients with sustained engraftment had an early spike of *Bacteroides* at 2 to 6 days post-FMT, which showed great variability among patients at subsequent time points (Fig. 5A); however, relative abundances of the predominant genera were generally stable throughout the year of follow-up. The decline in donor similarity in several patients corresponded with decreases in the abundance of the genera *Bacteroides* and *Parabacteroides* (Fig. 5B), both members of the phylum *Bacteroidetes*. Slower engraftment was similarly associated with a more gradual increase in the relative abundance of *Bacteroides* (Fig. 5C).

Spearman correlation analysis, done among all samples, further revealed significant positive correlations between the relative abundances of *Bacteroides*, *Parabacteroides*, or *Faecalibacterium* and percent donor similarity (Spearman’s ρ = 0.237, 0.373, and 0.239; *P* = 1.0 × 10^−4^ to 1.7 × 10^−2^). Pairwise differences in genus abundances, however, were not significant among the engraftment patterns at the same time point (*post hoc P* > 0.05). Similarly, differences in alpha diversity at the year time point did not vary significantly among the groups (*P* ≥ 0.464).

We further investigated population-level changes among members of the genus *Bacteroides* by using oligotype analysis. This analysis uses single nucleotide polymorphisms of 16S rRNA to identify likely strains within a target genus, offering greater resolution than that seen with OTU binning (21). Sixteen oligotypes were observed among all samples, with 10 oligotypes each observed in samples from donors 6 and 41, and six observed in samples from donor 44. Among all samples in which *Bacteroides* was detected, the donor samples harbored significantly more oligotypes than did the patient samples (*post hoc P* ≤ 0.003), and significantly fewer oligotypes were observed in pre-FMT samples relative to donors (*P* < 0.0001). Typically, oligotype diversity expanded in patient samples following cFMT, but usually only 2 to 4 engrafted (see Fig. S1 to S3 in the supplemental material). Changes in oligotype abundance had correspondence with the patients’ overall engraftment pattern, where divergence from the donor was associated with a reorganization of the oligotype assemblage or absence of members of the *Bacteroides*.

10.1128/mBio.01586-19.1 FIG S1 Bacteroides oligotypes among patients that showed sustained engraftment. The black line represents overall similarity to the donor sample. In samples in which oligotypes are absent but donor similarity is shown, *Bacteroides* were not detected. Similarly, patients not shown had a high frequency of *Bacteroides* non-detects among all samples. Download FIG S1, DOCX file, 0.6 MB. Copyright © 2019 Staley et al. 2019 Staley et al. This content is distributed under the terms of the Creative Commons Attribution 4.0 International license.

10.1128/mBio.01586-19.2 FIG S2 *Bacteroides* oligotypes among patients that showed declines of engraftment. The black line represents overall similarity to the donor sample. In samples in which oligotypes are absent but donor similarity is shown, *Bacteroides* were not detected. Similarly, patients not shown had a high frequency of *Bacteroides* non-detects among all samples. Download FIG S2, DOCX file, 0.2 MB. Copyright © 2019 Staley et al. 2019 Staley et al. This content is distributed under the terms of the Creative Commons Attribution 4.0 International license.

10.1128/mBio.01586-19.3 FIG S3 *Bacteroides* oligotypes among samples from R48, who showed slow engraftment. The black line represents overall similarity to the donor sample. In samples in which oligotypes are absent but donor similarity is shown, *Bacteroides* were not detected. Similarly, patients not shown had a high frequency of *Bacteroides* non-detects among all samples. Download FIG S3, DOCX file, 0.05 MB. Copyright © 2019 Staley et al. 2019 Staley et al. This content is distributed under the terms of the Creative Commons Attribution 4.0 International license.

## DISCUSSION

In this study, we tracked bacterial engraftment from encapsulated, freeze-dried donor preparations, delivered orally, through a year-long follow-up. Among patients who recovered from rCDI, the majority showed high levels of engraftment (≥70% similarity) after 1 week following cFMT, which was typically sustained throughout the year. This is similar to the previous report on 14 Finnish patients who received colonoscopic FMT, where recipient intestinal microbiota remained highly correlated (>90%) with that of the donor over 1 year (17). However, we (13) and others (15) also noted divergence of recipient fecal communities within the year following FMT. Moreover, a retrospective, longitudinal study of 93 rCDI patients receiving colonoscopic FMT observed that microbial communities in patients who responded to FMT showed greater differences in beta diversity than those patients who had recurrence (22). Importantly, we also noted differing patterns in both early and long-term engraftment stability following cFMT, although all patients analyzed showed clinical recovery. This finding suggests that donor similarity itself is not specifically related to clinical resolution of rCDI, and the FMT may simply act to reorganize the community to a healthy state.

The mechanism(s) by which FMT results in clinical recovery has only been partially elucidated (23) and likely involves changes in bile acid metabolism (24, 25), as well as reconstitution of alpha diversity that provides colonization resistance against C. difficile (22, 26). We previously noted that complete bacterial engraftment was not essential for recovery from rCDI by colonoscopic FMT (27). In this current study, we both provide a novel characterization of the long-term bacterial kinetics following encapsulated FMT and further demonstrate that neither complete nor sustained donor engraftment is necessary for long-term clinical recovery. A previous study in which a defined consortia of bacteria were transplanted into a C. difficile mouse model suggested that a seed bacterial community may serve as a scaffolding for further rearrangement of the intestinal microbiota, which eventually returned to a healthy state (28). While this idea is currently speculative, it is possible that a similar phenomenon results from cFMT administration, especially with the large number of microbial species being transplanted. However, this may be a rather simplistic understanding, and reconstitution of functional gut microbiota may also be governed by other ecological principles, including founder effects from initial colonizers (29); complex cooccurrence interactions (30); niche exclusion and inclusion, secondary successions, and hysteresis (31); and stochastic effects leading to new stable states.

Despite this complexity and while the level of donor similarity showed variation, other community parameters such as alpha diversity were consistent, despite the pattern of engraftment. Interestingly, genera within the *Bacteroidetes*, as well as the distribution of *Bacteroides* oligotypes, showed the greatest association with engraftment. Members of this genus were previously shown to be the primary component of the incremental response following cFMT (14) and were highly predictive of response or recurrence (18). Early *in vitro* studies reported that C. difficile inhibited the growth of *Bacteroides* spp. (32) and suggested that members of the genus *Bacteroides* contributed to resistance to C. difficile recurrence. It is also interesting that patients treated with fidaxomicin, an antibiotic used to treat C. difficile infection that spares *Bacteroidetes*, have lower rates of recurrence relative to the broader-spectrum drug vancomycin (33).

SourceTracker was originally developed to identify sources of contamination in metagenomic data sets (34) but was not intended to measure engraftment or transfer of highly similar communities. Use of SourceTracker to identify environmental sources of pollution has been robustly demonstrated (35), but its clinical relevance has not been rigorously tested. To validate SourceTracker results, we compared the percentage of the community comprised of shared OTUs between donor and patient samples. Shared OTUs represented a significantly greater extent of engraftment than that determined by SourceTracker, suggesting that the latter provides a conservative estimate of bacterial transfer between donor and patient. Moreover, compared to the artificial “combined” donor source, we observed a >20% increase in similarity among all patient samples, as we previously reported (18). These data suggest that post-FMT rearrangement of the microbiota due to external environmental variation may still reflect a shift toward a more healthy state, irrespective of divergence from donor. Nevertheless, at the 1-year time point, patient microbiota composition differed significantly based on the donor material that patients received, suggesting a sustained influence of the donor microbiota through the first year. Further study will be necessary to evaluate the duration of this donor-specific influence.

Two patients in the current cohort experienced recurrence of rCDI following cFMT and were excluded from the analysis. One patient, who received capsules from donor 41, had a series of post-FMT complications, including knee replacement, hospital-acquired pneumonia, progressive respiratory failure, heart attack, and coma in the months following cFMT. Together, these factors almost certainly compromised bacterial engraftment. The second patient, the recipient of capsules from donor 6, received antibiotics for treatment of a urinary tract infection (UTI) and was then administered methenamine hippurate (Hiprex) for UTI suppression, which may have also interfered with engraftment. Two patients (R67 and R68) received topical antibiotics following cFMT (metronidazole and clindamycin, respectively), which did not seem to have a significant impact on bacterial engraftment. Along these lines, two of three patients taking metformin, prior to and throughout the course of the study, showed considerably lower and delayed engraftment compared to others, while the third seemed unaffected. Metformin was previously shown to alter intestinal microbiota composition (36, 37). Future study will be necessary to further disentangle the effects of metformin on FMT engraftment.

Results of this study highlight different patterns of long-term bacterial engraftment following cFMT, exclusive of clinical recovery. Notably, taxonomic similarity to the donor did not seem to influence patient recovery, although an overall donor-specific influence on taxonomic composition separated patient communities up to a year following FMT. This study was limited by a small sample size, which may not have captured all possible post-FMT patterns of engraftment. We considered many clinical factors that may have significant impact on the composition of intestinal microbiome, including various medications, history of abdominal surgery (e.g., cholecystectomy), and metabolic factors such as obesity, all of which are encountered in this very complex patient population; however, their contribution could not be resolved given the study size. Also, we did not collect systematic dietary data following cFMT treatment. Further, our study focused only on bacterial community composition, although fungal and viral communities have also shown variable engraftment kinetics associated with FMT response (16, 38, 39). While bile acid metabolism has been related to cFMT response (14, 18), further mechanistic investigations will also be necessary to characterize functional changes and potential functional redundancies associated with the various efficacious reorganizations of the intestinal microbiota.

## MATERIALS AND METHODS

### Healthy stool donors.

All stool donors qualified in accordance with the strict inclusion and exclusion criteria described previously (8) and in accordance with the Investigational New Drug Application 15071 sponsored by the University of Minnesota Microbiota Therapeutics Program, which includes infectious disease, metabolic, and autoimmune testing. Exclusion criteria also included any history of gastrointestinal diseases or surgery, food intolerances or allergies, neurologic or psychiatric disorders, or history of antibiotic exposure within 6 months (none of the donors in the program have a history of antibiotic exposure within 3 years). The body mass index of all donors in the program is ≤25 kg m^−2^. Health care workers are excluded from the donor program because of the potential risk of colonization with multidrug-resistant organisms. The stool was tested for viral, bacterial, and parasitic enteric pathogens, as well as vancomycin-resistant enterococci, methicillin-resistant Staphylococcus aureus, carbapenem-resistant Enterobacteriaceae, and bacteria containing extended-spectrum beta-lactamases, which constitute part of the release criteria prior to clinical use. All donor activities, which include administration of questionnaires, physical exams, and laboratory testing, are approved by the University of Minnesota Institutional Review Board.

### Capsule preparation.

Capsules were prepared as previously described (11) using a single stool sample from each of three donors (donors 6, 41, and 44) enrolled in the University of Minnesota donor program. Briefly, fecal samples were homogenized by blending under N_2_ gas, sieved to remove large particles of >0.25 mm, amended with 5% trehalose, and freeze-dried. Each course of capsules represented only one fecal donation, and capsules were stored at −80°C prior to distribution to patients. Patients who received capsules from donor 6 took four capsules once representing a dose of 5 × 10^11^ cells, those who received capsules from donor 41 took two capsules once representing a dose of 2.1 × 10^11^ cells, and the patient who received capsules from donor 44 took four capsules once representing a dose of 2.5 × 10^11^. Differences in donor and dosage were previously reported not to affect cFMT efficacy (18).

### Patients and sample collection.

Detailed patient inclusion and exclusion criteria for our cFMT protocol were as described previously (11). Patients were enrolled if they had at least two prior recurrences of rCDI and were C. difficile toxin positive by PCR at least 3 months prior to treatment. Patients were administered oral vancomycin until 2 days prior to cFMT. Capsules were home delivered by a research coordinator and taken on an empty stomach, with only clear liquids for 2 h following. Patients remained in close contact with study and clinical staff throughout follow-up, and clinical recurrence was defined as a return of diarrheal symptoms and/or a positive toxin PCR result. Patients who experienced recurrence had the option of receiving a follow-up FMT by either capsule or colonoscopic delivery. This study was approved by the University of Minnesota Institutional Review Board, and all patients provided informed consent.

Fecal samples were collected in single-use toilet hats from which the patients transferred an aliquot (scoop) to a 30-ml polystyrene fecal specimen container (Globe Scientific, Inc., Paramus, NJ, USA). Samples were stored in the patients’ freezers prior to transfer on ice to the lab, where they were held at −20 to −80°C until DNA extraction. Sample collections were taken prior to cFMT (pre-FMT) and up to 409 days post-FMT. Due to the uneven nature of sample collection, samples were binned to the following day group ranges: 2 to 6 days, 7 to 21 days, 28 to 45 days, 86 to 134 days, and 346 to 409 days post-FMT, in correspondence with our previous studies (14, 18).

### DNA extraction and sequencing.

DNA was extracted from approximately 0.25-g aliquots of thawed stool samples by using the DNeasy PowerSoil extraction kit (Qiagen, Hilden, Germany) and the automated QIAcube platform (inhibitor removal technology [IRT] protocol). The V5-V6 hypervariable regions of the 16S rRNA gene were amplified using the BSF784/1064R primer set (40). Illumina adapters (Illumina Inc., San Diego, CA, USA) and barcodes were appended to amplicons using the dual-index method by the University of Minnesota Genomics Center (UMGC) (41), and paired-end sequencing was done on the Illumina MiSeq platform at a read length of 300 nucleotides (nt).

### Bioinformatics.

Sequence processing was done by using mothur software ver. 1.35.1 (42) and a previously published pipeline (18). Briefly, trimmed sequences were quality screened and aligned against the SILVA database ver. 132 (43). Operational taxonomic units (OTUs) were binned at 97% sequence similarity using the furthest-neighbor algorithm, and taxonomic classifications were made against the version 14 database from the Ribosomal Database Project (44). For unbiased statistical comparisons (45), the number of sequences per sample was rarefied to 11,000 reads by random subsampling.

SourceTracker ver 0.9.8 (34) was used with default parameters to predict the percentage of donor engraftment using (i) all three donor samples designated as a single source (“combined donor”) or (ii) only the donor lot that the patient received designated as the source (“individual donor”). To assess patterns in donor engraftment, SourceTracker predictions were normalized to pre-FMT samples by subtraction. OTUs predicted to be part of a source contribution were classified to genera for interpretation. For comparison, empirically shared OTUs were those found in both patient samples and the donor sample used to create the capsule lot that the patient received.

To further investigate changes in the *Bacteroides* populations of patients, oligotyping was done using version 2.1 software and suggested best practices (21). To be included in analyses, oligotypes had to have a minimum of 100 reads of a unique sequence, to account for at least 1% of sequence reads with a minimum abundance of 250 reads, and to occur in at least three patient samples. Twelve entropy points were used to differentiate oligotypes.

### Statistical analyses.

Good’s coverage estimate and the Shannon index (46) were calculated using mothur. Beta diversity was evaluated based on Bray-Curtis distances (47) using analysis of similarity (ANOSIM) (48), and ordination was performed by principal-coordinate analysis (49). Spearman correlations relating the relative abundances of genera and ordination position were determined using the corr.axes function in mothur. Differences in alpha diversity and engraftment were determined by ANOVA using Tukey’s *post hoc* test, and Spearman correlations were done to relate these two features. Statistics were calculated using XLSTAT ver. 17.06 (Addinsoft, Belmont, MA). All statistics were evaluated at α = 0.05, with Bonferroni correction for multiple comparisons.

### Data availability.

Raw sequence data are deposited in the Sequence Read Archive of the National Center for Biotechnology Information under BioProject accession number SRP070464.
