# Supplementary material for: Durable Long-Term Bacterial Engraftment following Encapsulated Fecal Microbiota Transplantation To Treat Clostridium difficile Infection
Source: mBio. 2019 Jul 23;10(4):e01586-19. doi: 10.1128/mBio.01586-19 (PMC6650559; doi:10.1128/mBio.01586-19)
Supplement: FIG S1 [file mBio.01586-19-sf001.docx]

**
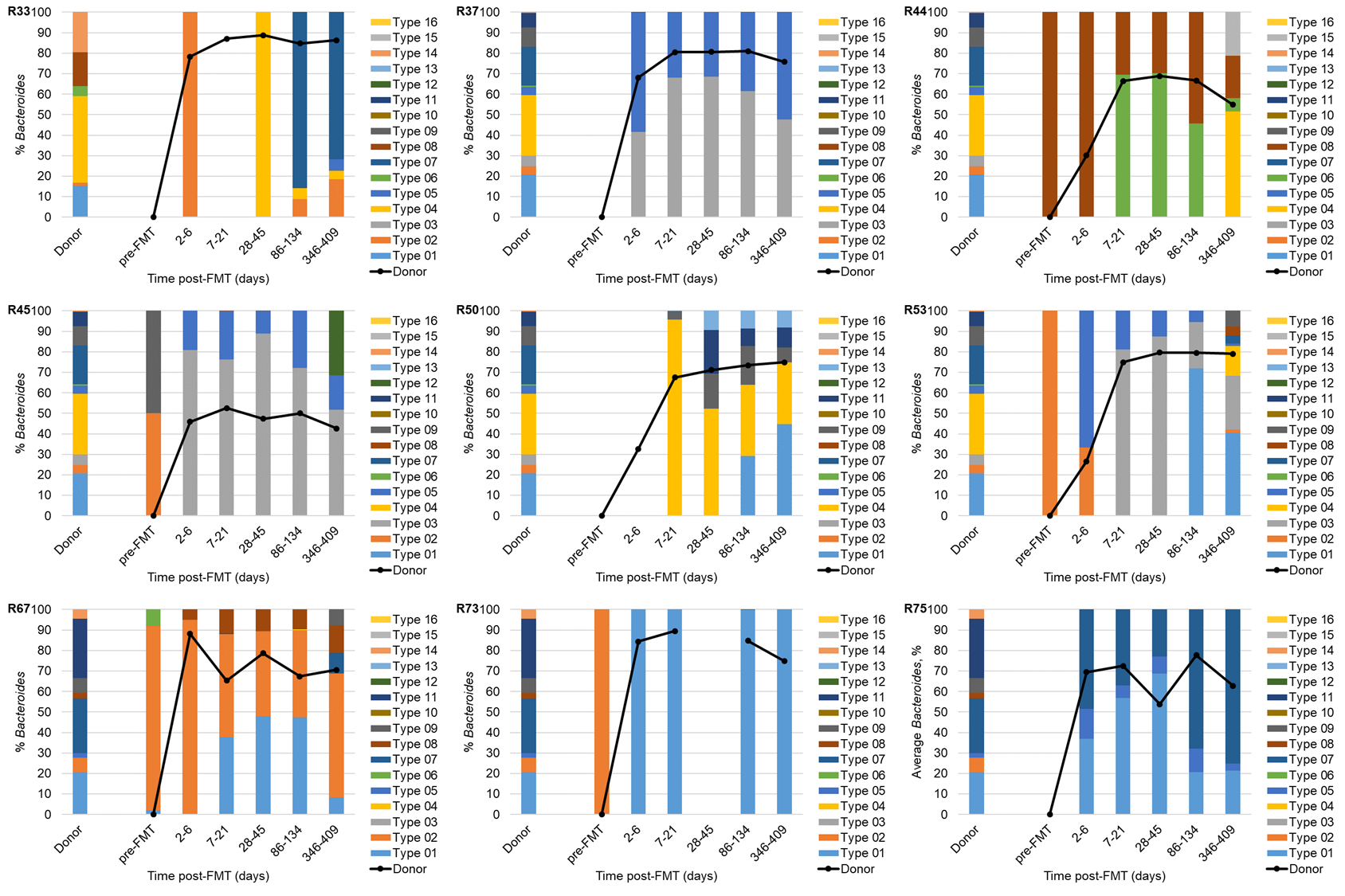
**

**Figure S1** – *Bacteroides* oligotypes among patients that showed sustained engraftment. The black line represents overall similarity to the donor sample. In samples in which oligotypes are absent but donor similarity is shown, *Bacteroides* were not detected. Similarly, patients not shown had a high frequency of *Bacteroides* non-detects among all samples.
