# Supplementary material for: Durable Long-Term Bacterial Engraftment following Encapsulated Fecal Microbiota Transplantation To Treat Clostridium difficile Infection
Source: mBio. 2019 Jul 23;10(4):e01586-19. doi: 10.1128/mBio.01586-19 (PMC6650559; doi:10.1128/mBio.01586-19)
Supplement: FIG S3 [file mBio.01586-19-sf003.docx]

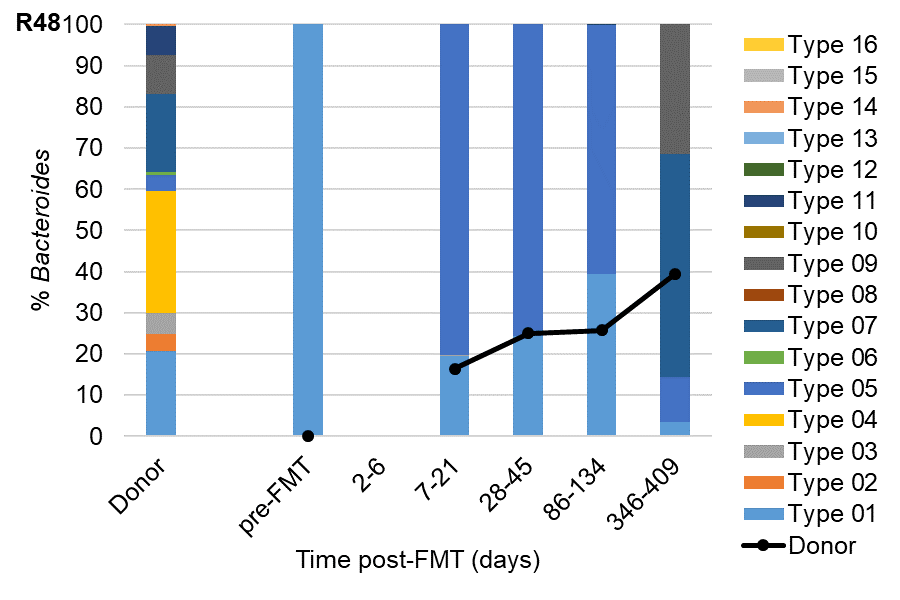


**Figure S3** - *Bacteroides* oligotypes among samples from R48, who showed slow engraftment. The black line represents overall similarity to the donor sample. In samples in which oligotypes are absent but donor similarity is shown, *Bacteroides* were not detected. Similarly, patients not shown had a high frequency of *Bacteroides* non-detects among all samples
